# Supplementary material for: Phenotypic and Functional Characterization of NK Cells in αβT-Cell and B-Cell Depleted Haplo-HSCT to Cure Pediatric Patients with Acute Leukemia
Source: Cancers (Basel). 2020 Aug 5;12(8):2187. doi: 10.3390/cancers12082187 (PMC7463940; doi:10.3390/cancers12082187)
Supplement: Supplementary file 1 [file cancers-12-02187-s001.pdf]

Supplementary Materials

Phenotypic and Functional Characterization of NK Cells in $\alpha\beta$T-Cell and B-Cell Depleted Haplo-HSCT to Cure Pediatric Patients with Acute Leukemia

Raffaella Meazza, Michela Falco, Fabrizio Loiacono, Paolo Canevali, Mariella Della Chiesa, Alice Bertaina, Daria Pagliara, Pietro Merli, Valentina Indio, Federica Galaverna, Mattia Algeri, Francesca Moretta, Natalia Colomar-Carando, Letizia Muccio, Simona Sivori, Andrea Pession, Maria Cristina Mingari, Lorenzo Moretta, Alessandro Moretta, Franco Locatelli, and Daniela Pende

A

	KIR-L		
	C1	C2	Bw4
Patient			
Mother			
Father			

B

	Inhibitory KIR					Activating KIR						KIR genotype	B content
	2DL1	2DL2	2DL3	3DL1	3DL2	2DS1	2DS2	2DS3	2DS4	2DS5	3DS1		
UPN 66 Mother				C					S4			B/X	3

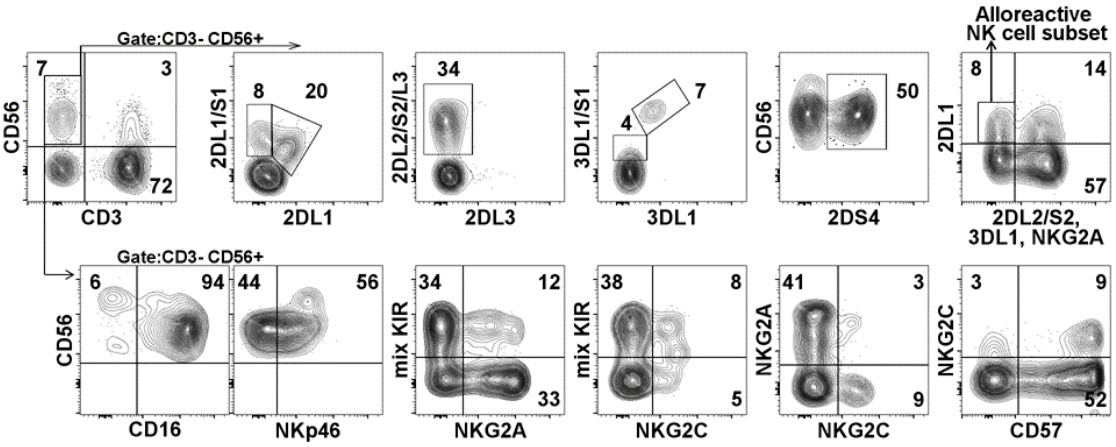

C

	Inhibitory KIR					Activating KIR						KIR	B
	2DL1	2DL2	2DL3	3DL1	3DL2	2DS1	2DS2	2DS3	2DS4	2DS5	3DS1	genotype	content
UPN 66 Father				C/T					S4/d			B/X	1

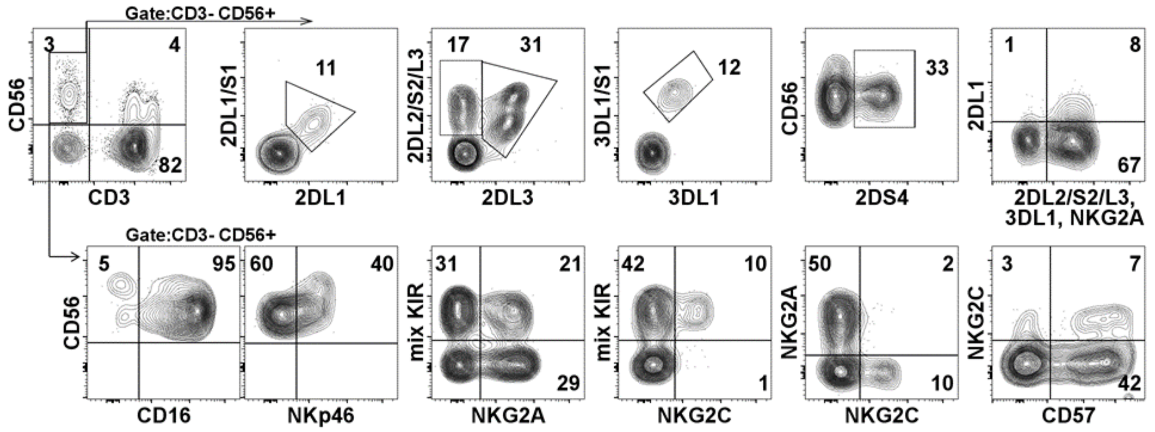

Figure S1. Genetic and phenotypic analyses of alternative donors. (**A**): Analysis of KIR-L of a recipient (UPN66) and possible donors (i.e., mother and father). The presence of KIR-L is indicated by colored boxes (gray and black) while white boxes represent the missing KIR-L. The black box indicates the mismatched KIR-L in the GvH direction (i.e., HLA-C2 in the mother). (**B**,**C**): *KIR* gene profile, *KIR* genotype, B content value of UPN66 mother (**B**) and father (**C**) are summarized in the tables. White boxes and colored boxes (gray and black) represent the absence and the presence of a KIR, respectively. "C" or "T" in *KIR3DL1* boxes indicate alleles coding for surface and intracellular retained receptors, respectively [1,2]. *KIR2DS4* alleles coding for surface receptors are reported as "S4", while those coding putative soluble molecules as "d". The educated iKIR relevant for the NK alloreactivity is indicated as a black box (i.e., KIR2DL1 in the mother). The analysis of the surface expression of inhibitory and activating receptors on NK cells (gating on $CD3^{-}CD56^{+}$ cells) from each donor is shown. The alloreactive NK cell subset has been also evaluated.

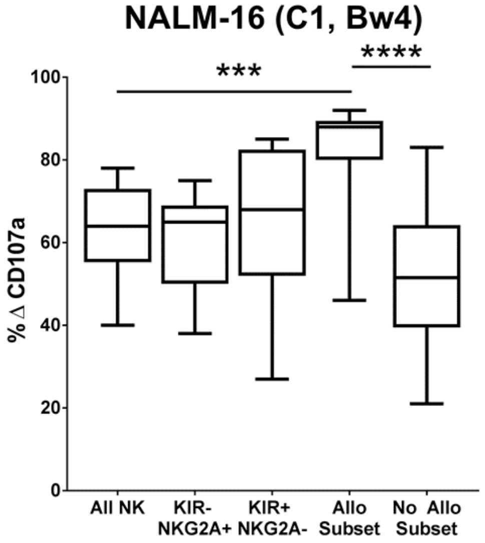

Figure S2. Degranulation capacity of NK cells stimulated by NALM-16 cell line. Different NK cell subsets derived from HLA-C1/C2 donors (n = 22) are compared for their degranulation activity (ΔCD107a) upon co-culture with NALM-16 cell line. E:T ratio 2:1. Whisker lines represent highest and lowest values; horizontal lines represent the median values. *** $p < 0.001$, **** $p < 0.0001$ (Kruskal-Wallis test with Dunn's post-test).

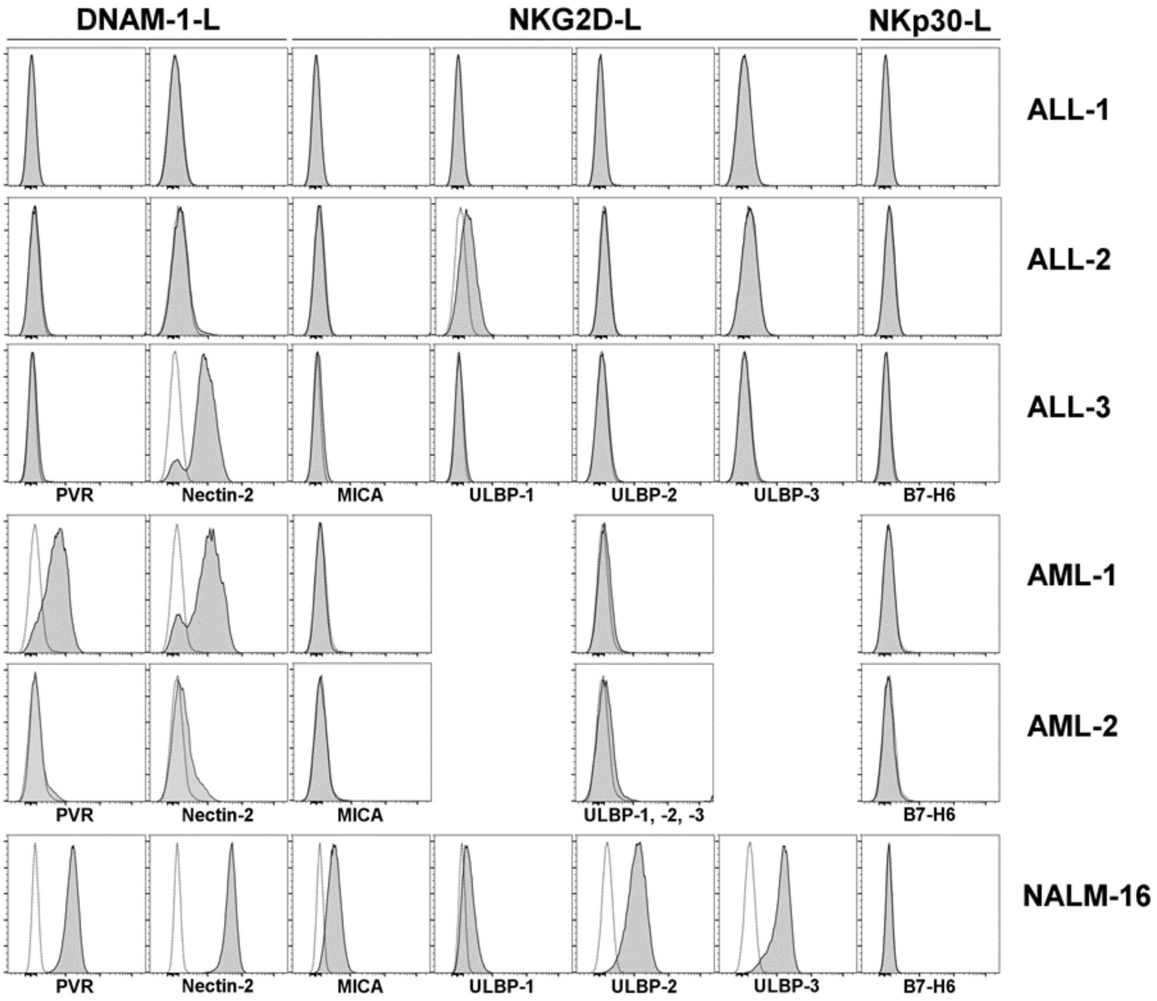

Figure S3. Expression of ligands for activating NK-cell receptors on leukemia cells. The expression of the ligands for the functionally investigated activating NK receptors on ALL or AML targets was investigated by indirect immunofluorescence and cytofluorimetric analysis using specific mAb and appropriate PE-conjugated isotype-specific secondary reagents (filled profiles). Empty profiles with dotted lines represent negative controls. For ULBP staining, either each mAb or pooled (ULBP-1, -2, -3) mAbs were used.

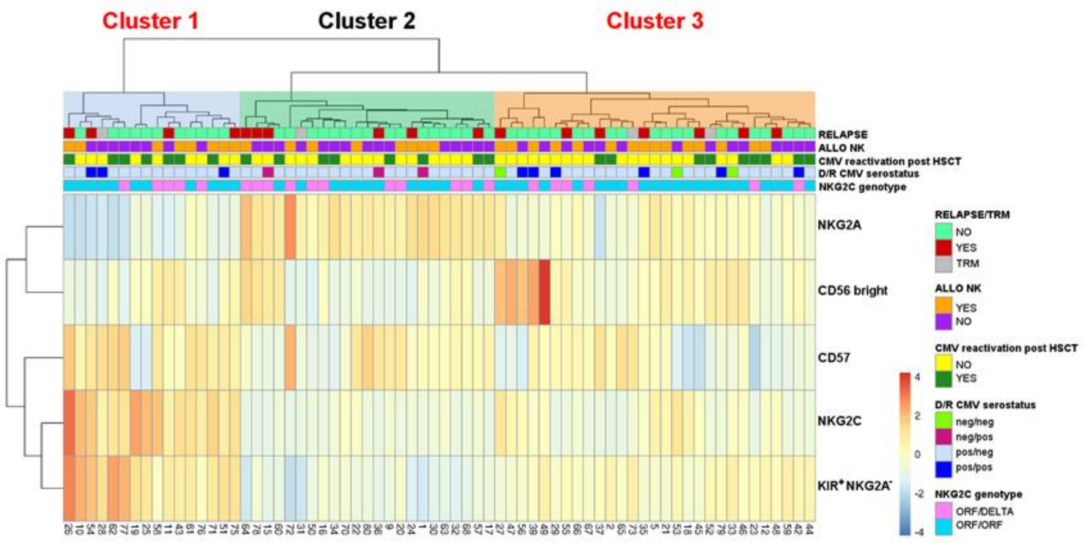

Figure S4. Unsupervised hierarchical clustering of NK cell phenotype of donors. Unsupervised hierarchical clustering of NK cell repertoires in 68 donors allowed the identification of three main clusters, namely cluster 1 (blue pattern) and cluster 3 (orange pattern) with "more differentiated" phenotypes, while cluster 2 (green pattern) with "naive" repertoire. Data were based on multi-color flow-cytometry, analyzing the frequencies of CD56^{bright}, or, among the CD56^{dim}, the frequencies of NKG2A^{+}, CD57^{+}, NKG2C^{+} and KIR^{+}NKG2A^{-} NK cells. Information on clinical and biological observations is annotated in the top bars above and to the right of the heat map. Z-scores normalization was computed and used to scale rows with the aim to highlight differences between samples. High (red) and low (blue) frequencies of each subset are represented in the color scale.

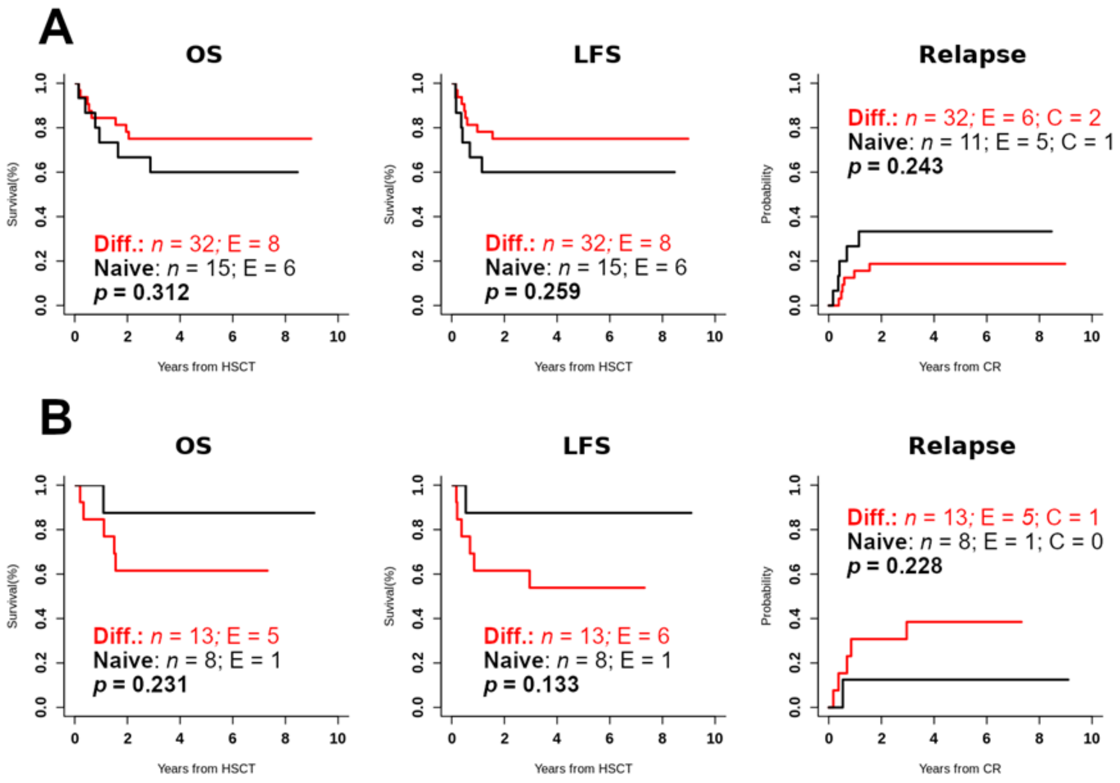

Figure S5. Correlation analysis of donor clusters with clinical outcome, stratifying by type of leukemia. The clusters identified in donors have been analyzed for possible association with OS, LFS, and cumulative incidence for Relapse in (**A**) ALL (n = 47) and in (**B**) AML (n = 21) patients. Fischer's exact text. The p values are indicated.

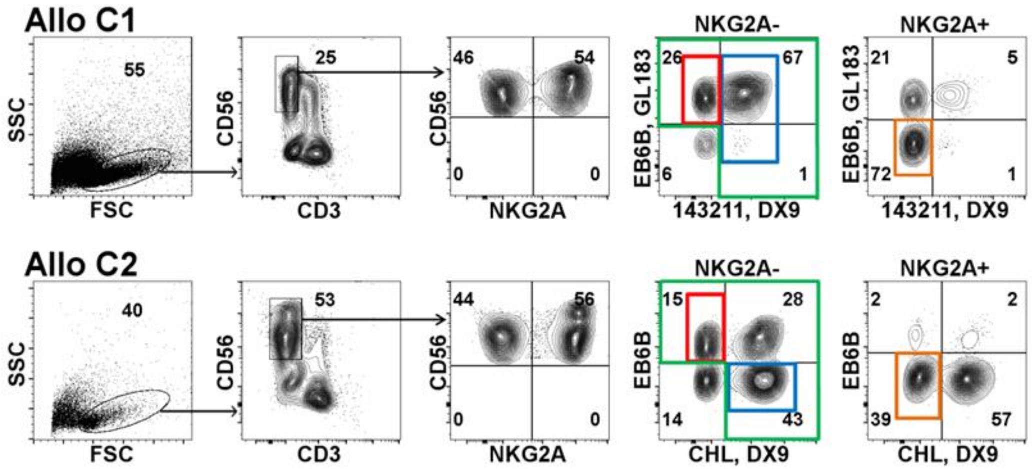

Figure S6. Gating strategy to define NK cell subsets in degranulation assays. The gating strategy to identify different NK cell subsets for degranulation assays of two representative post-transplant patients is shown. CD3$^-$CD56$^+$ NK cells can first be dissected into NKG2A$^-$ and NKG2A$^+$ cells. Among NKG2A$^-$ NK cells, appropriate anti-KIR mAb combinations allow to define the alloreactive subset (Allo Subset, red region), the non-alloreactive subset (No Allo Subset, blu region), the KIR$^+$NKG2A$^-$ subset (green region). Among the NKG2A$^+$ NK cells, orange region identifies the KIR$^-$NKG2A$^+$ subset.

Table S1. Patient and donor characteristics.

Disease (N° Cases)	NK Alloreactivity (N° cases)	2DS1 (E/U)	B/x *KIR* Genotype	B Content ≥ 2	Relapse	TRM
ALL (56)	Allo C1 (10)	5	9	4	3	1
	Allo C2 (8)	NA	5	2	0	0
	Allo Bw4 (6)	1	4	3	1	0
	No Allo (32)	13	27	15	9	2
AML (24)	Allo C1 (5)	3	4	2	2	0
	Allo C2 (6)	NA	4	2	2	0
	Allo Bw4 (1)	1	1	1	0	0
	No Allo (12)	5	10	7	2	1

Table S2. Characterization of pediatric leukemia blasts and cell line.

Leukemia Cells §	Type	KIR-L	HLA class I (Staining Index)		Ligands of Activating Receptors (POS/NEG)						
			HLA-I	HLA-C	DNAM-1-L		NKG2D-L				NKp30-L
			W6-32	DT9	PVR	Nectin-2	MICA	ULBP-1	ULBP-2	ULBP-3	B7-H6
ALL-1	T-ALL	C1/C1 ABw4	35.3	10.3	NEG	NEG	NEG	NEG	NEG	NEG	NEG
ALL-2	T-ALL	C2/C2 Bw4T, ABw4	83.9	4.4	NEG	NEG	NEG	POS	NEG	NEG	NEG
ALL-3	BCP-ALL	C1/C1	34.8	0.9	NEG	POS	NEG	NEG	NEG	NEG	NEG
AML-1	AML	C1/C1 Bw4I	44.6	1.4	POS	POS	NEG	NEG	NEG	NEG	NEG
AML-2	AML-M0	C1/C1 Bw4I	87.3	9.6	NEG	POS	NEG	NEG	NEG	NEG	NEG
NALM-16	BCP-ALL	C1 Bw4T	10.0	0.1	POS	POS	POS	POS	POS	POS	NEG

§ ALL-2 and AML-2 are primary leukemias derived from UPN54 and UPN27, respectively. The other leukemias have been obtained from pediatric patients not included in the cohort of this study.

Table S3. Antibodies used in immunofluorescence and flow cytometry.

Clone	Specificity	Fluorochrome	Supplier
UCHT1	CD3	PE-CF594, BV510	BD Bioscience, San Josè, CA USA
HIB19	CD19	PE-CF594	BD Bioscience, San Josè, CA USA
3G8	CD16	FITC	BD Bioscience, San Josè, CA USA
NCAM 16.2	CD56	BV421	BD Bioscience, San Josè, CA USA
CHL	KIR2DL2/S2/L3	FITC	BD Bioscience, San Josè, CA USA
H4A3	CD107a	PE	BD Bioscience, San Josè, CA USA
N901	CD56	PE-Cy7	Beckman Coulter, Brea, CA USA
EB6B	KIR2DL1/S1 and KIR2DL3*005	PE, PE-Cy7, APC	Beckman Coulter, Brea, CA USA
GL183	KIR2DL2/S2/L3	PE, PE-Cy7, APC	Beckman Coulter, Brea, CA USA
Z27	KIR3DL1/S1	PE, APC	Beckman Coulter, Brea, CA USA
FES172	KIR2DS4	APC	Beckman Coulter, Brea, CA USA
Z199	NKG2A	PE, APC	Beckman Coulter, Brea, CA USA
BW264/56	CD3	VioBlue	Miltenyi Biotech, Bergisch Gladbach Germany
5B1	CD45	APC-Vio770	Miltenyi Biotech, Bergisch Gladbach Germany
9E2	NKp46 (CD335)	PE	Miltenyi Biotech, Bergisch Gladbach Germany
DX9	KIR3DL1	FITC, PE-Vio770	Miltenyi Biotech, Bergisch Gladbach Germany
REA110	NKG2A	FITC	Miltenyi Biotech, Bergisch Gladbach Germany
134591	NKG2C	AlexaFluor488	R&D systems, Minneapolis, MN USA
143211	KIR2DL1, 2DS5	FITC, PE	R&D systems, Minneapolis, MN USA
HCD57	CD57	Pacific Blue	Biolegend, San Diego, CA USA
ECM 41	KIR2DL3 (no *005)	Unconjugated	Our laboratory [3,4]
TU145	CD48	IgM	BD Bioscience, San Josè, CA USA
W6/32	HLA-class I	IgG2a	Our Laboratory
DT9	HLA-C	IgG2b	Millipore-Merck, Milan (ITALY)
BAM195	MIC-A	IgG1	Our Laboratory [5]
M295	ULBP-1	IgG1	Kindly provided by Amgen, Los Angeles, CA USA [5]
M311	ULBP-2	IgG1	Kindly provided by Amgen, Los Angeles, CA USA [5]
165903	ULBP-2	IgG2a	R&D systems, Minneapolis, MN USA
166510	ULBP-3	IgG2a	R&D systems, Minneapolis, MN USA
L14	Nectin-2 (CD112)	IgG2a	Our Laboratory [6]
L95	PVR (CD155)	IgG1	Our Laboratory [6]
17B1.3	B7-H6	IgG1	Kindly provided by E. Vivier, Marseille, France [7]

Table S4. Antibody combinations used to define Alloreactive NK cell subsets.

Type of Alloreactivity #	Permissive iKIR	Antibody Combinations*	
		PE-Conjugated	FITC-Conjugated
Allo C1	KIR2DL2/L3	GL183§	143211, DX9, NKG2A
Allo C2	KIR2DL1	EB6B	CH-L, DX9, NKG2A
Allo Bw4	KIR3DL1	Z27§	143211, CH-L, NKG2A

Defined as KIR-L present in the donor and absent in the recipient. * Appropriate fluorochrome–conjugated anti-CD3 and anti-CD56 mAb combinations were also used to identify NK cells in PBMC. § In KIR2DS1$^+$ donors, EB6B-PE was also added to include, in the alloreactive NK cell subset, this aKIR. The size of the alloreactive NK cell subset is calculated as the percentage of PE-positive and FITC-negative cells. In the presence of KIR2DS2, Allo C2 and Bw4 subsets can be under-estimated since there is no mAb capable to distinguish KIR2DL2/L3 from KIR2DS2.

Table S5. Antibody combinations used to define Alloreactive NK cell subsets in degranulation assays.

Type of Alloreactivity	Permissive iKIR	Antibody Combinations*		
		PC7-Conjugated	FITC-Conjugated	APC-Conjugated
Allo C1	KIR2DL2/L3	GL183[§]	143211, DX9	NKG2A
Allo C2	KIR2DL1	EB6B	CH-L, DX9	NKG2A

[§] In KIR2DS1^{+} donors, EB6B-PC7 was also added to include, in the alloreactive NK cell subset, this aKIR. The alloreactive NK cell subset is defined as APC-negative, PC7-positive and FITC-negative cells, as reported in Figure S6.

References

1. Pando, M.J.; Gardiner, C.M.; Gleimer, M.; McQueen, K.L.; Parham, P. The protein made from a common allele of KIR3DL1 (3DL1*004) is poorly expressed at cell surfaces due to substitution at positions 86 in Ig domain 0 and 182 in Ig domain 1. *J Immunol* **2003**, *171*, 6640–6649.
2. Alicata, C.; Pende, D.; Meazza, R.; Canevali, P.; Loiacono, F.; Bertaina, A.; Locatelli, F.; Nemat-Gorgani, N.; Guethlein, L.A.; Parham, P., et al. Hematopoietic stem cell transplantation: Improving alloreactive Bw4 donor selection by genotyping codon 86 of KIR3DL1/S1. *European journal of immunology* **2016**, *46*, 1511–1517, doi:10.1002/eji.201546236.
3. Vitale, M.; Carlomagno, S.; Falco, M.; Pende, D.; Romeo, E.; Rivera, P.; Della Chiesa, M.; Mavilio, D.; Moretta, A. Isolation of a novel KIR2DL3-specific mAb: comparative analysis of the surface distribution and function of KIR2DL2, KIR2DL3 and KIR2DS2. *Int Immunol* **2004**, *16*, 1459–1466, doi:10.1093/intimm/dxh147.
4. Falco, M.; Romeo, E.; Marcenaro, S.; Martini, S.; Vitale, M.; Bottino, C.; Mingari, M.C.; Moretta, L.; Moretta, A.; Pende, D. Combined genotypic and phenotypic killer cell Ig-like receptor analyses reveal KIR2DL3 alleles displaying unexpected monoclonal antibody reactivity: identification of the amino acid residues critical for staining. *J Immunol* **2010**, *185*, 433–441, doi:10.4049/jimmunol.0903632.
5. Pende, D.; Rivera, P.; Marcenaro, S.; Chang, C.C.; Biassoni, R.; Conte, R.; Kubin, M.; Cosman, D.; Ferrone, S.; Moretta, L., et al. Major histocompatibility complex class I-related chain A and UL16-binding protein expression on tumor cell lines of different histotypes: analysis of tumor susceptibility to NKG2D-dependent natural killer cell cytotoxicity. *Cancer Res* **2002**, *62*, 6178–6186.
6. Bottino, C.; Castriconi, R.; Pende, D.; Rivera, P.; Nanni, M.; Carnemolla, B.; Cantoni, C.; Grassi, J.; Marcenaro, S.; Reymond, N., et al. Identification of PVR (CD155) and Nectin-2 (CD112) as cell surface ligands for the human DNAM-1 (CD226) activating molecule. *J Exp Med* **2003**, *198*, 557–567, doi:10.1084/jem.20030788.
7. Brandt, C.S.; Baratin, M.; Yi, E.C.; Kennedy, J.; Gao, Z.; Fox, B.; Haldeman, B.; Ostrander, C.D.; Kaifu, T.; Chabannon, C., et al. The B7 family member B7-H6 is a tumor cell ligand for the activating natural killer cell receptor NKp30 in humans. *J Exp Med* **2009**, *206*, 1495–1503, doi:10.1084/jem.20090681.

© 2020 by the authors. Licensee MDPI, Basel, Switzerland. This article is an open access article distributed under the terms and conditions of the Creative Commons Attribution (CC BY) license (http://creativecommons.org/licenses/by/4.0/).
